# Research in Cerebrospinal Fluid Leak (Rhinorrhea and Otorrhea): A Bibliometric Analysis From 1945 to 2018

**DOI:** 10.7759/cureus.21888

**Published:** 2022-02-03

**Authors:** Jose-Manuel Ramos-Rincon, Irene Monjas-Canovas, Javier Abarca-Olivas, Juan-Ramón Gras-Albert, Isabel Bellinchón-Romero, Gregorio Gonzalez-Alcaide

**Affiliations:** 1 Department of Clinical Medicine, Miguel Hernandez University, Alicante, ESP; 2 Department of Otorhinolaryngology, General University Hospital of Alicante, Alicante, ESP; 3 Department of Neurosurgery, General University Hospital of Alicante, Alicante, ESP; 4 Department of History of Science and Documentation, University of Valencia, Valencia, ESP

**Keywords:** cerebrospinal fluid otorrhea, cerebrospinal fluid rhinorrhea, cerebrospinal fluid leak, publications, bibliometric

## Abstract

Objective

In this study, we aimed to analyze research activity on cerebrospinal fluid (CSF) leaks in general and CSF rhinorrhea and otorrhea in particular and to identify the main topic clusters in these areas.

Methods

We identified all relevant documents, using the medical subject heading (MeSH) term “Cerebrospinal Fluid Leak”, that are indexed in the MEDLINE database between 1945 and 2018. We performed a descriptive bibliometric analysis and analyses of networks and research clusters in order to identify the main topic areas of research.

Results

From 1945 to 2018, a total of 4,130 records were published with the term CSF leak, including 2,821 documents (68.1%) with the term CSF rhinorrhea and 1,040 documents (25.8%) with CSF otorrhea. The number of documents published increased from 10 in 1945-49 to 642 in 2010-14. Articles represented the dominant document type (86.8% of the documents analyzed), while case reports were the main type of study (37.4%). In terms of geographical distribution, researchers from the USA led in the number of signatures (39.1%), followed by those from the UK (7.5%). The most active areas of research in the field were “Postoperative Complications,” “Tomography, X-Ray Computed,” and “Magnetic Resonance Imaging.” The terms “Adults,” “Young Adult,” and “Middle-Aged” were most common in CSF rhinorrhea research; and the terms “Infant,” “Child, Preschool,” “Child,” and “Adolescent” were more common in CSF otorrhea.

Conclusions

Based on our findings, articles and case reports related to “Surgery” and “Postoperative Complications” associated with the diagnosis are the main topics of study, highlighting the importance of this document type in advancing knowledge in the field.

## Introduction

Cerebrospinal fluid (CSF) leak is the discharge of CSF through a hole in the skull bone, most commonly involving drainage from the nose (CSF rhinorrhea) or from the ear (CSF otorrhea), occurring either through the external auditory meatus or through the eustachian tube into the nasopharynx [1]. Common etiologies of CSF rhinorrhea include trauma, neoplasms, and prior surgery, while CSF otorrhea is usually associated with craniocerebral trauma, neurosurgical procedures, or other conditions; however, both conditions may occur spontaneously [2].

The past decades have witnessed a significant increase in research publications in the field of otorhinolaryngology [3]. Bibliometrics has been established as a discipline enabling the analysis of the scientific activity in a particular field or area of knowledge through the quantification of the bibliographic characteristics of scientific research [4]. Several studies have analyzed the scientific production in the fields of otorhinolaryngology and head-neck surgery [5-7] and neurosurgery [8-15]. Moreover, there are several publications focusing specifically on the topic in different countries, including African countries, and specifically Morocco [9,11]. Other manuscripts have analyzed specific research topics in neurosurgery, like central nervous system arteriovenous malformations [8], idiopathic intracranial hypertension [10], endoscopic third ventriculostomy [12], carotid artery stenting [13], pituitary adenoma [14], and craniopharyngioma [15].

One of the areas that have seen significant development within bibliometrics is the generation of visual representations, networks, and maps of scientific activity. These have great analytic potential, helping to characterize the structures, groupings, and interconnections between the elements under analysis, including the descriptors assigned to the documents, such as in the present study. Although bibliometric indicators have been used to approach the study of numerous diseases [4,16], this methodology has not been applied to research on the well-known condition of CSF leak, which encompasses surgical, diagnostic, and therapeutic-radiological aspects, among others.

The aim of this study is to describe the research production on CSF leak through bibliometric indicators, using the documents indexed in the MEDLINE database between 1945 and 2018. We analyze the evolution in the number of publications, the publication types, the document categories of clinical interest, the journals of publication, the geographical distribution of the research, and the topics addressed according to the different types of CSF leak (CSF rhinorrhea and CSF otorrhea) by means of co-word maps.

## Materials and methods

Identification of documents on “Cerebrospinal Fluid Leak”

The first step in this study was to identify the documents about the CSF leak in the MEDLINE database, the main database of reference for researchers and professionals in the health sciences. It is an open-access resource that permits, through the use of the medical subject headings (MeSH) thesaurus, the precise identification of documents that address the concepts under analysis as well as the contents of those documents. The CSF leak descriptor was included in 2005 in the database. This term encompasses the specific terms “Cerebrospinal Fluid Rhinorrhea” and “Cerebrospinal Fluid Otorrhea,” both included in 1966. The search of indexed documents with any of these three descriptors was carried out on the Clarivate Analytics Web of Science (WOS) platform, which includes the MEDLINE database, on January 21, 2019.

The Clarivate Analytics WOS platform provides information on the institutional affiliation of all authors. Using this strategy, 4,155 documents were retrieved, but we excluded 25 editorials, leaving 4,130 documents to be analyzed. The analysis of the scientific production by country was limited to the 3,019 documents (73.1%) included in the WOS database.

Standardization of bibliographic information

Using the bibliographic information from the retrieved documents, we created a relational database to standardize the bibliographic data and calculate the study variables as follows:

Publication Type [Field: Publication Type (PT)]

Based on the information collected from this field, we classified the records according to the document types (articles, reviews, and letters) as well as the clinical approach (case reports, clinical trials, controlled clinical trials, evaluation studies, meta-analysis, observational studies, practice guidelines, randomized clinical trials, and validation studies).

Institutional Affiliation of Papers Included in WOS [Field: Author Address (C1)]

This field includes information relating to the institutional affiliation of the authors signing the documents. Although all institutional affiliations have been available for papers published in MEDLINE since 2014, we aimed to study and present the information using a standardized approach, and hence we limited the analysis to the papers included in the WOS.

Descriptors [Fields: MeSH Terms (MH) and MeSH Subheadings (Also Called Qualifiers) (SH)]

We differentiated the MeSH terms from the subheadings or topic qualifiers (82 auxiliary descriptors that specify the aspect being addressed in relation to the MeSH terms) and identified the descriptors assigned with respect to CSF leak, CSF rhinorrhea, and CSF otorrhea.

Calculation of indicators

To analyze the scientific activity of the area, we determined the number of documents published each year, the journal of publication, the document types, the clinical approach to the study, and the countries of the authors signing the documents. This information generates a picture of the scientific activity, the media used (scientific journals), and the weight of countries leading the research in general and according to the different types of CSF (rhinorrhea or otorrhea).

To characterize the research in the area at a topic level, we determined the frequency with which the descriptors assigned to the documents appeared for each group: the generic CSF leak group and the specific document groups on CSF rhinorrhea and CSF otorrhea. We constructed topic maps to analyze the relationship between the descriptors and the research groups around them, that is, the existing research clusters, approaches, and specializations. This process involved the following steps:

Determination of the Co-occurrence of the Descriptors Assigned to the Documents and Generation of a Matrix of Absolute Values

The joint assignment of two descriptors in a single document implies a thematic affinity, as both aspects are addressed simultaneously in the same paper

Elimination of Generic Descriptors

We eliminated some excessively generic descriptors that did not describe relevant information on the content or that presented a high density of relationships with the rest of the descriptors.

Visual Representation of the Network

To establish the existing topic clusters in the area and represent them visually, we used a clustering algorithm in the VOSViewer program, which helps to detect the communities (clusters) within a network, made up of groups of homogeneous items that are strongly related to each other [17].

Statistical analysis

We compared the MeSH terms in CSF rhinorrhea- and CSF otorrhea-related documents using the Chi-squared test. P-values of less than 0.05 were considered statistically significant. We used SPSS Statistics version 22.0 (IBM, Armonk, NY) for statistical analysis.

Ethical considerations

Due to the nature of the study and dataset, it was not necessary to obtain informed consent or approval from an institutional ethics committee.

## Results

Evolution of production

**Figure 1:**
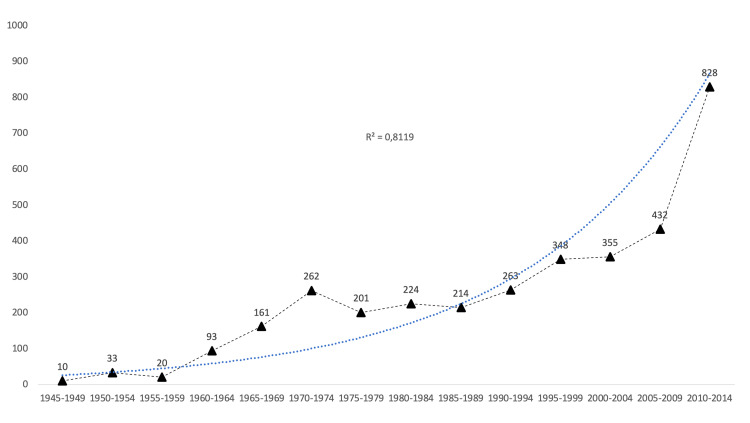
Evolution of the number of documents published on CSF leak by five-year periods The number of documents indexed in the 2015-2018 period was 686 (lower as it is a four-year period and due to time lags in indexing the documents of the most prolific period) CSF: cerebrospinal fluid

From 1945 to 2018, 4,130 articles were published involving CSF leak terms. The term CSF rhinorrhea was found in 2,821 documents (68.1%) and CSF otorrhea in 1,040 (25.8%). Both terms were present in 316 documents, while 269 documents used only the generic CSF leak descriptor. Figure 1 shows the evolution of the number of documents published on CSF leak.

Publication type distribution

The main publication type was journal article, which comprised 86.8% (n=3,584) of the documents; followed at a considerable distance by reviews, which made up 9.4% (n=390); and letters at 3.8% (n=156). With regard to the type of clinical research, 37.4% (n=1,544) of the documents were case reports; 1.7% (n=74) were clinical trials; 0.6% (n=48) were evaluation studies; and 0.5% (n=19) were meta-analyses. The rest of the study types were negligible in number (10 observational studies, eight validation studies, and two practice guidelines).

Journal distribution and thematic categories

The documents were published in 735 different journals. The main journals were World Neurosurgery/Surgical Neurology (n=178), Laryngoscope (n=176), Journal of Neurosurgery (n=134), Otology & Neurotology/American Journal of Otology (n=124), and Otolaryngology-Head and Neck Surgery (n=101). These five journals published 16.8% of the documents. Moreover, there were 371 journals with just one document, which cumulatively represented 9.0% of the total documents. Table 1 presents the details of the top 17 journals with at least 1.0% of the total publications on CSF leak. About three-quarters of the documents (n=3,019, 73.1%) were published in journals included in the WOS Journal Citation Reports. The three main WOS categories were Otorhinolaryngology (29.3%) Surgery (20.7%), and Clinical Neurology (19.8%), followed by Medicine; General and Internal (5.9%); and Radiology, Nuclear Medicine and Medical Imaging (5.2%).

**Table 1:** The 17 top-ranking journals (≥1% research production) in research production on CSF leak *World Neurosurgery was called Surgical Neurology until 2010. **Otology & Neurotology was called American Journal of Otology until 2001. ***Impact Factor from 2009 NI: not included in Journal Citation Reports; CSF: cerebrospinal fluid

Top Journals	Number of Documents	% of Documents	Impact Factor, 2017	Journal Category (Ranking, 2017)
World Neurosurgery/Surgical Neurology*	178	4.3	1.924	Clinical Neurology (139/194); Surgery (95/200)
Laryngoscope	176	4.1	2.442	Medicine. Research and Experimental Medicine (73 of 233): Otorhinolaryngology (12 of 41)
Journal of Neurosurgery	134	3.1	4.319	Clinical Neurology (37/197); Surgery (14/200)
Otology & Neurotology/American Journal of Otology**	124	3.0	2.182	Clinical Neurology (121/197); Otorhinolaryngology (13/41)
Otolaryngology-Head and Neck Surgery	101	2.3	2.444	Otorhinolaryngology (11/41); Surgery (67/200)
Journal of Laryngology and Otology	88	2.0	0.967	Otorhinolaryngology (36/41)
Acta Neurochirurgica	86	2.0	1.834	Clinical Neurology (146/197); Surgery (106/200)
Neurosurgery	85	2.0	4.475	Clinical Neurology (36/197); Surgery (12/200)
Journal of Craniofacial Surgery	57	1.3	0.785	Surgery (177/200)
British Journal of Neurosurgery	53	1.2	1.481	Clinical Neurology (171/197); Surgery (140/200)
Otolaryngologic Clinics of North America	50	1.2	1.514	Otorhinolaryngology (21/41)
Annals of Otology, Rhinology, and Laryngology	49	1.1	1.458	Otorhinolaryngology (22/41)
International Journal of Pediatric Otorhinolaryngology	47	1.1	1.305	Otorhinolaryngology (34/41); Pediatrics (87/124)
No Shinkei Geka. Neurological Surgery	43	1.0	0.131***	Neuroscience (146/197); Surgery (106/200)
HNO	43	1.0	0.914	Otorhinolaryngology (38/41)
Ear, Nose & Throat Journal	43	1.0	1.010	Otorhinolaryngology (35/41)
Annales d'Oto-laryngologie et de Chirurgie Cervico-faciale	43	1.0	NI	

Geographical distribution

Of the 3,019 articles included in WOS, 2,578 had information on the country of the institution producing the manuscript. Table 2 shows the number of documents published on CSF leak by the 15 most productive countries, both by total and types of CSF. Only 160 of the 2,578 documents with country data (6.21%) showed evidence of international collaboration. The leading country in total scientific production on CSF, as well as specific production on CSF rhinorrhea and CSF otorrhea, was the USA, followed by the UK. Other countries with significant research contributions were Japan, Germany, Italy, and France, with little difference between the number of documents on CSF rhinorrhea and that on CSF otorrhea.

**Table 2:** Top 15 countries producing research on CSF leak, CSF rhinorrhea, and CSF otorrhea (1945-2018) - included in the Web of Science and providing institutional data *The totals in this column include all documents that have been assigned one of the following MeSH: Cerebrospinal fluid leak (without specifying the type of drainage), Cerebrospinal Fluid Otorrhea, or Cerebrospinal Fluid Rhinorrhea CSF: cerebrospinal fluid; MeSH: medical subject headings

All Documents* (n=2,578)				CSF Rhinorrhea (n=1,619)			CSF Otorrhea (n=585)		
Country	Number of Documents	% of Documents	Country		Number of Documents	% of Documents	Country	Number of Documents	% of Documents
USA	1,007	39.06	USA		612	37.8	USA	245	41.8
UK	193	7.49	UK		118	7.29	UK	61	10.43
Japan	166	6.44	Germany		113	6.98	Germany	46	7.86
Germany	142	5.51	Japan		86	5.31	Japan	28	4.79
Italy	128	4.96	Italy		83	5.13	France	26	4.44
France	110	4.27	France		79	4.88	Italy	18	3.08
China	101	3.92	Turkey		71	4.38	Canada	16	2.73
Turkey	99	3.84	India		64	3.95	South Korea	13	2.22
India	87	3.37	China		47	2.9	Switzerland	12	2.05
Canada	63	2.44	Switzerland		42	2.59	Turkey	12	2.05
South Korea	60	2.33	Canada		40	2.47	China	12	2.05
Spain	57	2.21	Spain		38	2.35	Spain	11	1.88
Switzerland	54	2.09	Australia		35	2.16	India	11	1.88
Australia	46	1.78	South Korea		32	1.98	Israel	11	1.88
Brazil	44	1.71	Brazil		29	1.79	Australia	10	1.71

MeSH distribution

The three principal MeSH terms in the field were “Postoperative Complications,” “Tomography, X-Ray Computed,” and “Magnetic Resonance Imaging.” MeSH terms that were significantly more common in documents relating to CSF rhinorrhea compared to CSF otorrhea were “Magnetic Resonance Imaging,” “Treatment Outcome,” “Endoscopy,” “Dura Mater,” “Neurosurgical, Procedures,” “Pituitary Neoplasms,” and “ Sphenoid Sinus.” The terms “Meningitis,” “Recurrence,” “Ear, Inner,” “Neuroma, Acoustic,” “Mastoid,” and “Otitis Media” appeared more frequently in relation to CSF otorrhea (Table 3).

**Table 3:** Top descriptors (MeSH) and qualifiers assigned to papers on CSF leak, CSF rhinorrhea, and CSF otorrhea (1945-2018) *The totals in this column include all documents that have been assigned one of the following MeSH: Cerebrospinal fluid leak (without specifying the type of drainage), Cerebrospinal Fluid Otorrhea, or Cerebrospinal Fluid Rhinorrhea CSF: cerebrospinal fluid; MeSH: medical subject headings

	All Documents*		CSF Rhinorrhea		CSF Otorrhea		P-value
	Number of Documents	% of Documents	Number of Documents	% of Documents	Number of Documents	% of Documents	
MeSH Terms							
Postoperative Complications	1,008	24.41	645	22.86	220	21.15	0.28
Tomography, X-Ray Computed	912	22.08	637	22.58	226	21.73	0.62
Magnetic Resonance Imaging	585	14.16	377	13.36	107	10.29	0.012
Treatment Outcome	582	14.09	369	13.08	99	9.52	0.003
Endoscopy	572	13,85	486	17.23	22	2.12	<0.001
Skull Fractures	414	10.02	343	12.16	131	12.60	0.87
Meningitis	401	9.71	293	10.39	142	13.65	0.001
Skull Base	368	8.91	267	9.46	131	12.60	0.005
Fistula	351	8.50	269	9.54	92	8.85	0.55
Dura Mater	292	7.07	208	7.37	32	3.08	<0.001
Neurosurgical Procedures	290	7.02	192	6.81	17	1.63	<0.001
Pituitary Neoplasms	280	6.78	233	8.26	12	1.15	<0.001
Sphenoid Sinus	248	6.00	233	8.26	6	0.58	<0.001
Encephalocele	240	5.81	169	5.99	64	6.15	0.76
Craniocerebral Trauma	226	5.47	184	6.52	77	7.40	0.37
Recurrence	215	5.21	137	4.86	79	7.60	0.001
Ear, Inner	157	3.80	35	1.24	127	12.21	<0.001
Neuroma, Acoustic	149	3.61	97	3.44	69	6.63	<0.001
Mastoid	106	2.57	24	0.85	83	7.98	<0.001
Otitis Media	86	2.08	7	0.25	80	7.69	0.001
Qualifiers							
Surgery	2,540	61.5	1,758	62.32	566	54.42	<0.001
Etiology	2,444	59.18	1,622	57.5	631	60.67	0.082
Complications	1,502	36.37	1,035	36.69	410	39.42	0.13
Diagnosis	1,226	29.68	858	30.41	358	34.42	0.019
Methods	1,195	28.93	809	28.68	189	18.17	<0.001
Diagnostic Imaging	954	23.1	645	22.86	220	21.15	0.52
Adverse Effects	727	17.6	441	15.63	136	13.08	0.21
Pathology	683	16.54	437	15.49	162	15.58	0.89
Injuries	504	12.2	377	13.36	120	11.54	0.43
Therapy	432	10.46	251	8.9	103	9.9	0.89
Prevention & Control	385	9.32	254	9	102	9.81	0.97
Therapeutic Use	347	8.4	225	7.98	100	9.62	0.62
Epidemiology	293	7.09	148	5.25	68	6.54	0.14
Abnormalities	292	7.07	142	5.03	149	14.33	<0.001

Qualifiers

The most common qualifiers were “Surgery” and “Etiology.” The qualifiers “Surgery” and “Methods” were significantly more common in CSF rhinorrhea, whereas the qualifier “Abnormalities” was significantly more common in CSF otorrhea (Table 3).

Generic MeSH terms

Table 4 shows the frequency of MeSH terms referring to human/animal research, female/male, age groups, and type of study, in all CSF documents and by type of CSF. Most of the research was performed on humans. The proportion of documents relating to males and females was similar in CSF rhinorrhea and CSF otorrhea. In terms of age, the terms “Infant,” “Newborn,” “Adults,” “Young Adult,” and “Middle-Aged” were more common in CSF rhinorrhea; and the terms “Infant,” “Child, Preschool,” “Child,” and “Adolescent” were significantly more common in CSF otorrhea. The most common MeSH terms describing the type of study were “Retrospective Studies” and “Follow-Up Studies,” with similar results in both types of CSF.

**Table 4:** Distribution of MeSH terms referring to the type of research, gender, age group, and type of study on CSF leak, CSF rhinorrhea, and CSF otorrhea (1945-2018) *The totals in this column include all documents that have been assigned one of the following MeSH: Cerebrospinal fluid leak (without specifying the type of drainage), Cerebrospinal Fluid Otorrhea, or Cerebrospinal Fluid Rhinorrhea CSF: cerebrospinal fluid; MeSH: medical subject headings

MeSH Terms	All Documents*		CSF Rhinorrhea		CSF Otorrhea		P-value
	Number of Documents	% of Documents	Number of Documents	% of Documents	Number of Documents	% of Documents	
Type of Research							
Humans	4,067	98.47	2,781	98.58	1,029	98.94	0.42
Animals	67	1.62	41	1.45	11	1.06	0.42
Gender							
Female	2,316	56.08	1,598	56.65	576	55.38	0.52
Male	2,397	58.04	1,549	54.91	533	51.25	0.20
Age Group							
Infant, Newborn	48	1.16	118	4.18	18	1.73	<0.001
Infant	249	6.03	118	4.18	125	12.02	<0.001
Child, Preschool	455	11.02	250	8.86	210	20.19	<0.001
Child	728	17.63	453	16.06	288	27.69	<0.001
Adolescent	835	20.22	578	20.49	246	23.65	0.023
Adult	1,869	45.25	1,351	47.89	379	36.44	<0.001
Young Adult	317	7.67	187	6.63	34	3.27	<0.001
Middle-Aged	1,816	43.97	1,280	45.37	355	34.13	<0.001
Aged	985	23.85	636	22.55	230	22.12	0.80
Aged, 80 and Over	243	5.88	140	4.96	46	4.42	0.93
Type of Study							
Retrospective Studies	760	18.4	451	15.99	157	15.10	0.53
Follow-Up Studies	405	9.81	272	9.64	96	9.23	0.61
Cohort Studies	53	1.28	32	1.13	7	0.67	0.21

Visual representation of the network of MeSH terms

**Figure 2:**
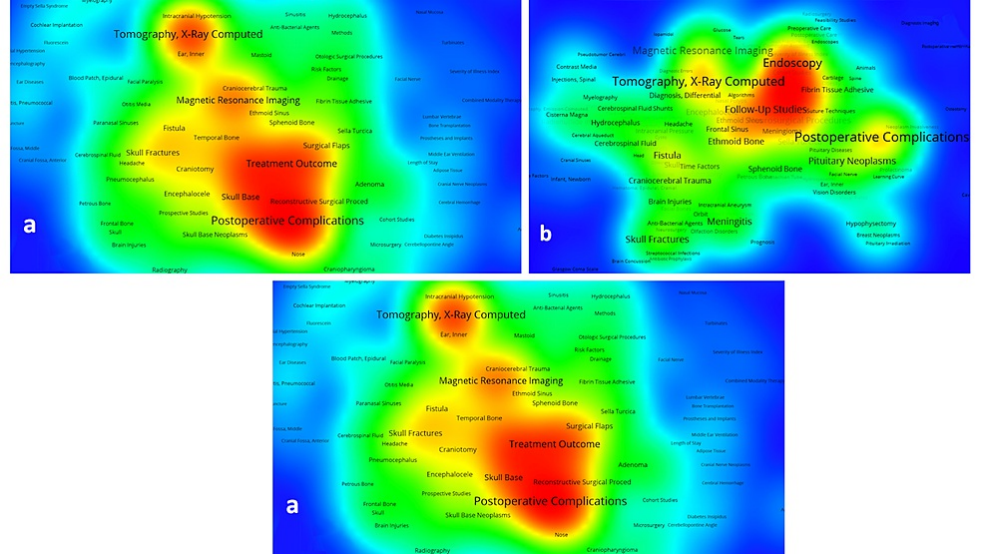
Analysis of topic clusters according to MeSH assigned to publications (a) on CSF leak, (b) CSF rhinorrhea, and (c) CSF otorrhea The different groupings, in the form of “islands” in red tones, represent the main clusters of the thematic networks, while the chromatic gradation illustrates the areas with a lower density of relations between the MeSH in yellow and green tones. The spatial distribution of the MeSH and their proximity to each other respond to the intensity of co-occurrence between them CSF: cerebrospinal fluid; MeSH: medical subject headings

Figure 2 presents the visual representation of the network of MeSH terms.

## Discussion

The number of articles published on CSF leak steadily increased throughout the study period, which is normal, as most bibliometric analyses on other diseases also reveal an upward trend in the number of publications [18-20]. The most significant aspect to point out is the predominance of case reports (approximately 35%), which show similar values for other surgical areas as well, such as maxillofacial surgery (31%) [21]. Individually, case reports are an important source of clinical information but they only provide partial grounds on which to base treatment decisions; but when they are considered together and adequately codified and integrated into structured information systems, they can provide early insight into characterizing rare diseases, as they allow physicians to compare cases and check diagnoses [4].

At a journal level, this study shows the important multidisciplinary approach in the area, as the three most prominent publications were journals of otorhinolaryngology, surgery, and clinical neurology. The multidisciplinary approach in science is a tradition that has been associated with greater advances in knowledge, translation of results, and impact of research [16,22].

USA was the predominant country in research production, followed by various European countries (UK, Germany, France, and Italy) and Japan. This fact also applies specifically to the otolaryngology field [5,7], neurosurgery [8,10,12], as well as other domains in health sciences [18,19]. The top countries in research production did not include any of the African nations, while the only Latin American country featured was Brazil. The top-contributing Asian countries (in addition to Japan) were India, China, and South Korea, while Turkey was ranked eighth. A combination of factors can explain these observations. First of all, the USA's dominance in scientific production related to CSF leak reflects its global leadership in all spheres of scientific research. Furthermore, the need to have a surgical structure to produce research in this field would favor the most developed countries to a greater extent [6,7,14,15]. The level of international collaboration was very low (6.21%), which highlights the need to develop surgical structures that favor the promotion of research in countries with less scientific development and implement strategies that favor multidisciplinary collaboration [22].

With regard to the MeSH terms, CSF rhinorrhea was the main branch, while surgery and postoperative complications were the most important sub-topics. Relevant topics related to managing patients with CSF were otorhinolaryngology and clinical neurology [1]. Research also focused on identifying risk factors and assessing different treatments and their outcomes, as shown by the terms related to etiology (“Skull Fracture” of “Skull Base”), diagnosis (especially via “Tomography, X-Ray Computed” or “Magnetic Resonance Imaging”), treatment (“Treatment Outcome” or “Endoscopy”), and complications (meningitis).

There is a difference in the patient profile of research between CSF rhinorrhea and CSF otorrhea, with the former area concentrating on older patients (“Adult” or “Middle-Aged”). Spontaneous CSF rhinorrhea is associated with increased intracranial pressure and is considered a manifestation of idiopathic intracranial hypertension in middle-aged people, whereas secondary CSF rhinorrhea is associated with trauma in the same age group [23,24]. On the other hand, CSF otorrhea research focused on the “Infant,” “Child, Preschool,” “Child” and “Adolescent” age groups, reflecting the fact that CSF otorrhea can be primary and in most cases is secondary to pediatric skull base fractures18. Thus, even though CSF rhinorrhea and CSF otorrhea are included under the same umbrella MeSH term, the profile of research is quite different between these sub-fields.

The main limitation of this study was that we did not analyze the citations, with a focus on the journals with the highest impact and dissemination at an international level; this perspective would be necessary to reach a truly comprehensive view of research in the field. Likewise, other aspects could also be considered, such as co-authorship networks or gender disparities in scholarly productivity [7].

## Conclusions

The present study on CSF leak-related scientific production has revealed some features that differ notably from bibliometric analyses on other clinical pathologies: the predominant interest in surgery and the weightage of clinical case reports, which emerge as the primary channel for generating and disseminating knowledge in the field. Postoperative complications, diagnostic aspects related to CT and MRI, treatment outcomes, and surgical procedures such as endoscopy were also topics of interest. The USA leads the research production in CSF leak, while European countries and emerging countries such as China have contributed less when compared to other research areas.
